# Modified Medial Para-Olecranon Pinning Versus Conventional Crossed Pinning for Displaced Pediatric Supracondylar Humerus Fractures: A Retrospective Comparative Cohort Study

**DOI:** 10.3390/children13081063

**Published:** 2026-08-10

**Authors:** Hassan Salah Ibrahim, Abdulla Abdelwahab, Girgis Saad, Habib Al Ismaily

**Affiliations:** Department of Orthopedics and Trauma Surgery, Rashid Hospital, Mohammed Bin Rashid University of Medicine and Health Sciences, Dubai 505055, United Arab Emirates; asabdelwahab@dubaihealth.ae (A.A.); gslatif@dubaihealth.ae (G.S.); hmalismaily@dubaihealth.ae (H.A.I.)

**Keywords:** pediatric supracondylar humerus fracture, crossed pinning, medial pinning, percutaneous fixation, Kirschner wire, ulnar nerve injury, pediatric trauma, Flynn criteria

## Abstract

**Highlights:**

**What are the main findings?**

- Modified medial para-olecranon pinning was associated with favorable Flynn functional outcomes compared with conventional crossed pinning.
- The modified technique was associated with shorter operative time and fewer observed postoperative ulnar nerve injuries while maintaining comparable fracture union and radiographic alignment.

**What are the implications of the main findings?**

- The technique may represent a practical alternative medial entry strategy for surgeons who prefer crossed-pin fixation in selected displaced pediatric supracondylar humerus fractures.
- Prospective multicenter clinical studies and dedicated anatomical investigations are required before broader recommendations can be made.

**Abstract:**

**Background/Objectives**: Closed reduction and percutaneous pinning is the standard surgical treatment for displaced pediatric supracondylar humerus fractures. Although crossed-pin fixation provides excellent biomechanical stability, medial pin insertion remains associated with the risk of iatrogenic ulnar nerve injury. The modified medial para-olecranon technique has been introduced as an alternative medial wire insertion strategy while preserving the principles of crossed-pin fixation. This study compared its clinical performance with conventional crossed pinning. **Methods**: A retrospective comparative cohort study was conducted at a tertiary referral trauma center between January 2017 and December 2024. Seventy children younger than 14 years with Gartland type II–IV supracondylar humerus fractures met the inclusion criteria. All patients treated with the modified medial para-olecranon technique (*n* = 35) were included. A comparison cohort of 35 patients treated with conventional crossed pinning was selected from 68 eligible conventionally treated patients by computer-generated random sampling stratified by Gartland type (frequency matching), after application of identical eligibility criteria. The primary outcome was functional outcome assessed using the Flynn criteria. Secondary outcomes included operative time, radiographic alignment, fracture union, and postoperative complications. **Results**: Baseline demographic and fracture characteristics were comparable between groups. Mean age was 6.7 ± 2.8 years in the modified medial para-olecranon group and 6.9 ± 2.6 years in the conventional crossed-pin group. The modified medial para-olecranon group had a lower mean Flynn score (6.80 ± 3.31 vs. 10.74 ± 3.70; mean difference, −3.94; 95% CI, −5.61 to −2.27; *p* < 0.001) and a higher proportion of excellent Flynn outcomes (77.1% vs. 28.6%). Operative time was shorter in the modified medial para-olecranon group (47.49 ± 7.50 vs. 66.00 ± 8.19 min; mean difference, −18.51 min; 95% CI, −22.26 to −14.76; *p* < 0.001). Fracture union occurred at a comparable time in both groups (27.71 ± 3.94 vs. 28.34 ± 4.12 days; mean difference, −0.63 days; 95% CI, −2.55 to 1.29), and postoperative Baumann angles were similar. No postoperative iatrogenic ulnar nerve injuries occurred in the modified medial para-olecranon group, whereas two occurred in the conventional crossed-pin group; both were transient sensory paraesthesia that resolved without exploration within six months. Overall complications occurred in 1 patient (2.9%) and 7 patients (20.0%), respectively (odds ratio, 0.17; 95% CI, 0.03 to 1.02; Fisher exact *p* = 0.055). **Conclusions**: In this retrospective comparative cohort, the modified medial para-olecranon technique was associated with favorable functional outcomes, shorter operative time, and fewer observed postoperative ulnar nerve injuries, although the difference in complications did not reach statistical significance, while maintaining comparable radiographic alignment and fracture healing. Because treatment was not randomized and potential confounders could not be adjusted for, these between-group differences should be interpreted as associations rather than as evidence of a causal treatment effect. Further prospective multicenter studies and dedicated anatomical investigations are required before broader recommendations can be made.

## 1. Introduction

Supracondylar humerus fractures are the most common elbow fractures in children and one of the most frequent pediatric injuries requiring urgent operative fixation. They account for a substantial proportion of pediatric elbow fractures and occur most often in early school-age children after a fall onto an outstretched hand. Although most children recover well after appropriate treatment, displaced fractures can be associated with malunion, cubitus varus, elbow stiffness, neurovascular injury, compartment syndrome, and functional limitation when reduction or fixation is inadequate [1,2,3].

Displaced type II, III, and multidirectionally unstable type IV fractures commonly require closed reduction and percutaneous Kirschner-wire fixation [4,5,6]. The goal of surgical treatment is restoration of both radiographic reduction and functional elbow alignment and motion.

Despite broad agreement regarding operative fixation of displaced fractures, the ideal pin configuration remains debated. Crossed medial–lateral pinning has strong biomechanical support because it provides greater resistance to rotational and translational displacement than many lateral-only constructs, particularly in unstable fracture patterns [7,8,9,10]. This mechanical advantage explains why crossed pinning remains widely used despite increasing interest in lateral-entry fixation.

The principal concern with crossed-pin fixation is the risk of iatrogenic ulnar nerve injury during medial pin insertion. Systematic reviews and comparative studies have shown that medial pin placement is associated with a higher rate of ulnar nerve palsy than lateral-only constructs, although reported rates vary according to technique, elbow position, fracture pattern, surgeon experience, and whether a mini-open medial approach is used [11,12,13,14]. This complication is clinically important because even transient nerve dysfunction causes concern for patients, families, and surgeons, and persistent palsy may require exploration or further treatment.

Several strategies have therefore been described to improve the safety of medial pin insertion while preserving the mechanical advantages of a crossed-pin construct. These include placing the medial pin with the elbow less flexed or extended, direct palpation of the medial epicondyle, use of a mini-open medial approach, and other technical modifications intended to reduce the risk of nerve injury during medial wire passage [15,16,17,18,19]. Although each method has potential advantages, none has eliminated variation in surgical practice, and comparative clinical data evaluating alternative medial entry points remain limited. Although medial para-olecranon and other modified medial entry points have been described in technical reports, to our knowledge they have not been directly compared with conventional crossed pinning in a clinical cohort, which is the gap the present study addresses.

Anatomical variability further complicates the issue. The position of the ulnar nerve around the medial elbow is not uniform in all children, and dynamic imaging studies have shown that its relationship to the medial epicondyle can change unpredictably with elbow flexion [20,21]. Therefore, a technique should not be assumed to protect the nerve solely on the basis of a proposed anatomical mechanism unless that mechanism is directly evaluated. Clinical outcome studies can report observed complications, but they cannot establish the spatial relationship between the wire and the ulnar nerve without dedicated imaging, anatomical, or intraoperative assessment.

Most previous comparative studies have focused on crossed pinning versus lateral-only fixation rather than on modifications of medial pin entry. Lateral-entry pinning may reduce the risk of medial nerve injury, but crossed-pin fixation continues to be preferred by many surgeons for selected unstable fractures because of its mechanical properties [22,23,24,25,26,27]. Consequently, there remains a practical clinical need for medial pinning strategies that preserve crossed-pin stability while minimizing complications associated with medial wire insertion.

The modified medial para-olecranon technique evaluated in the present study changes the medial wire entry point while preserving the principles of crossed-pin fixation and bicortical purchase. The present investigation was designed to evaluate the clinical performance of this technique rather than to prove an anatomical mechanism.

The primary objective was to compare functional and radiographic outcomes of the modified medial para-olecranon technique with conventional crossed pinning in children with displaced supracondylar humerus fractures. We hypothesized that the modified technique would maintain comparable fracture union and radiographic alignment while being associated with favorable functional outcomes, shorter operative time, and fewer observed postoperative ulnar nerve injuries.

## 2. Materials and Methods

### 2.1. Study Design

This retrospective comparative cohort study was conducted at Rashid Hospital, Dubai, United Arab Emirates, a tertiary referral trauma center. Pediatric patients who underwent operative treatment for displaced supracondylar humerus fractures between January 2017 and December 2024 were included. The study was approved by the University Student and Resident Research Subcommittee (USRRSC), a subcommittee under the Mohammed Bin Rashid University of Medicine and Health Sciences Institutional Review Board (MBRU-IRB) (protocol code MBRU IRB-2024-750; approval date: 1 May 2025). Research site approval was granted on 18 November 2024. Owing to the retrospective design and use of anonymized institutional data, the requirement for individual informed consent was waived. The study was conducted in accordance with the principles of the Declaration of Helsinki and was reported in accordance with the STROBE guidelines.

### 2.2. Patient Selection

**Figure 1:**
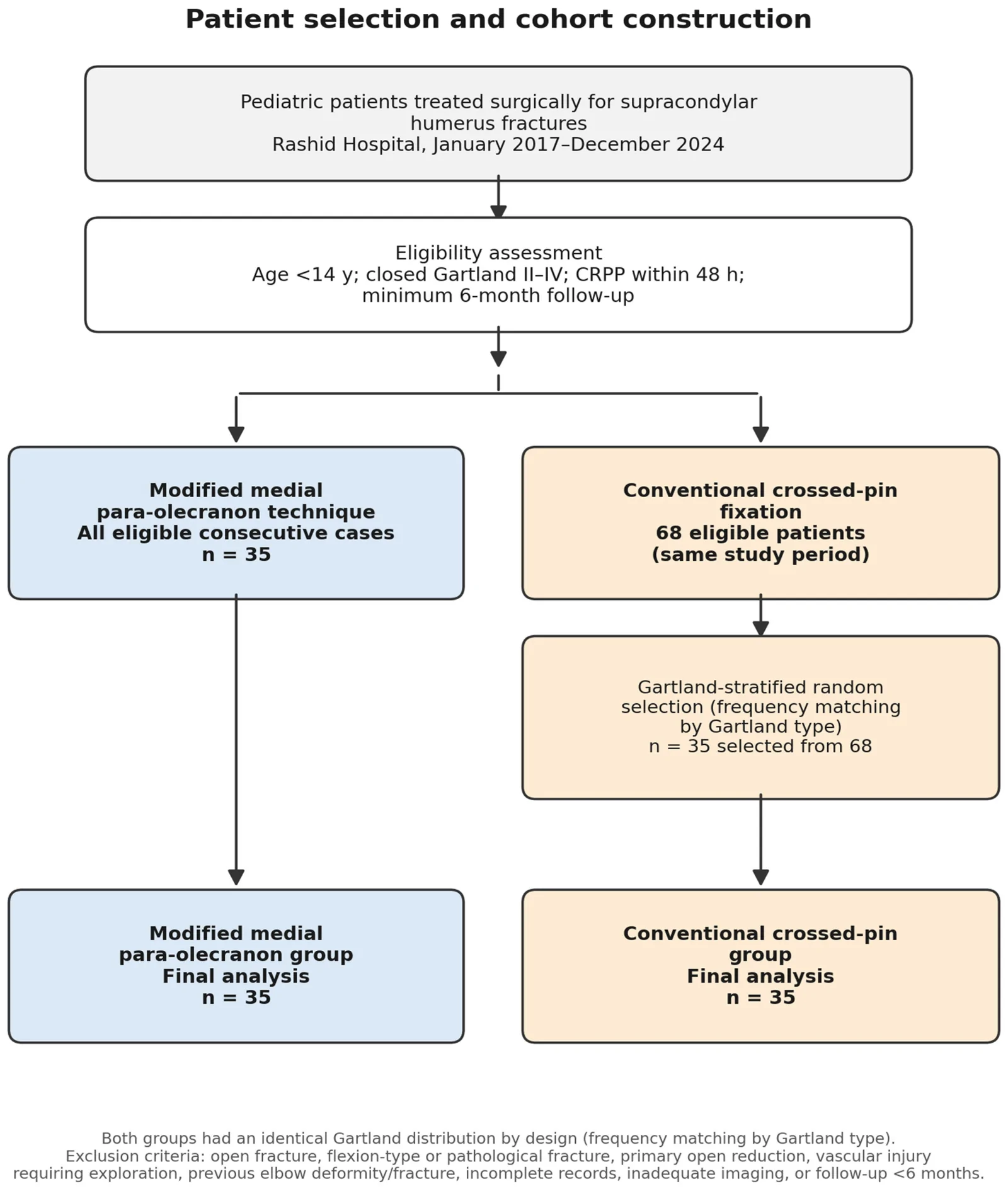
STROBE-style patient flow diagram. All eligible consecutive modified medial para-olecranon cases were included (*n* = 35). From 68 eligible conventional crossed-pin patients, 35 were selected by Gartland-stratified computer-generated random sampling (frequency matching by Gartland type), producing an identical Gartland distribution in the two groups.

Patients were identified from the institutional electronic medical record system (EPIC/SALAMA) and Picture Archiving and Communication System (PACS). All consecutive patients treated using the modified medial para-olecranon technique during the study period who fulfilled the eligibility criteria were included (*n* = 35). During the same period, 68 patients underwent conventional crossed-pin fixation and met the identical eligibility criteria, forming the control pool. To obtain a comparable control cohort while minimizing selection bias, the 68 eligible patients were stratified by Gartland type, and 35 were then selected by computer-generated random sampling within each Gartland stratum, so that the control group’s Gartland distribution matched that of the modified para-olecranon group (frequency matching by Gartland type). Allocation to the modified or conventional technique was determined by individual surgeon preference and expertise rather than by fracture characteristics or randomization. Within the conventional group, the decision to add a mini-open medial exposure was likewise made by the treating surgeon rather than by protocol; patients were not randomized to treatment, and only selection of the control cohort was randomized. Frequency matching by Gartland type balances fracture severity across groups and accounts for their identical Gartland distribution; however, because allocation to the two techniques was itself based on surgeon preference, matching does not remove confounding by indication. The study selection process is illustrated in Figure 1.

### 2.3. Inclusion and Exclusion Criteria

Patients were eligible if they were younger than 14 years; had a closed Gartland type II, III, or IV supracondylar humerus fracture; underwent closed reduction and percutaneous pinning within 48 h of injury; and had at least six months of clinical and radiographic follow-up. Patients were excluded if they had open fractures, flexion-type fractures, pathological fractures, previous fracture or deformity of the injured elbow, vascular injury requiring immediate exploration, primary open reduction, incomplete records, inadequate radiographs, or follow-up shorter than six months.

### 2.4. Surgical Technique

**Figure 2:**
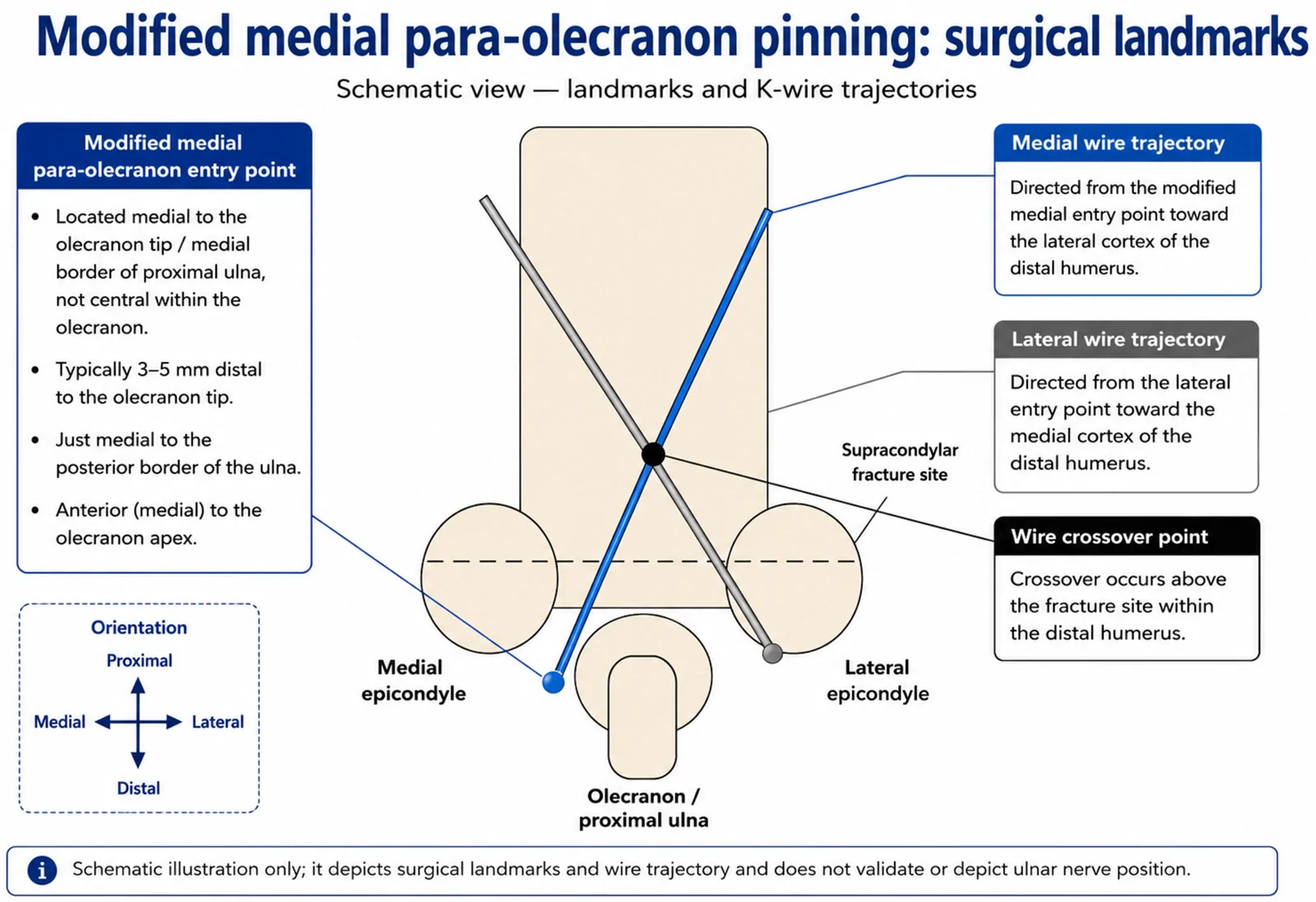
Schematic illustration of the modified medial para-olecranon pinning technique. The figure shows the intended modified medial para-olecranon entry point relative to the olecranon/proximal ulna and the medial and lateral Kirschner-wire trajectories. In the schematic, the blue line indicates the modified medial (para-olecranon) Kirschner wire, the grey line indicates the lateral Kirschner wire, and the dashed line indicates the supracondylar fracture. The schematic illustrates surgical landmarks and wire trajectory only; it does not depict or validate ulnar nerve position.

All procedures were performed under general anesthesia using fluoroscopic guidance by consultant orthopedic trauma surgeons experienced in pediatric fracture fixation; three consultants performed the modified medial para-olecranon technique and three performed conventional crossed pinning, according to individual surgeon preference and expertise. Because all operating surgeons were experienced consultants, learning-curve effects are unlikely to account for the observed differences, although surgeon-level differences in technique and case selection cannot be excluded. Closed reduction was achieved according to standard principles based on fracture displacement, and reduction quality was confirmed fluoroscopically before fixation. In the modified medial para-olecranon technique, fracture alignment was maintained with the elbow in hyperflexion. A lateral Kirschner wire was inserted first through the lateral epicondyle. A second wire was then introduced through a medial para-olecranon entry point approximately 1–2 mm medial to the tip of the olecranon and in line with the tip in the proximal–distal axis (neither proximal nor distal to it). This wire was inserted percutaneously and advanced across the fracture under fluoroscopic guidance to obtain bicortical fixation. The modified medial para-olecranon entry point and intended K-wire trajectories are illustrated schematically in Figure 2. Wire position, divergence, bicortical purchase, and reduction were confirmed using anteroposterior, lateral, and oblique fluoroscopic views. The study evaluated clinical performance of this entry point and did not directly investigate ulnar nerve anatomy. In the conventional crossed-pin group, the lateral wire was inserted first, followed by a medial wire through the medial epicondyle according to the treating surgeon’s routine practice. In the conventional group, medial pinning required partial elbow extension in all 35 cases, which could transiently challenge maintenance of the hyperflexed reduction. A mini-open medial exposure to identify and protect the ulnar nerve before medial wire insertion was used at the discretion of the treating surgeon in 10 of the 35 conventional cases (28.6%); the remaining 25 cases (71.4%) were performed percutaneously. By contrast, the modified para-olecranon technique was performed percutaneously with the elbow kept in hyperflexion in all 35 cases, which helped hold the reduction. Because the conventional technique required elbow extension in every case and additionally involved a mini-open exposure in a subset of patients, whereas the modified technique was uniformly percutaneous and performed in hyperflexion, the two approaches differed in more than the medial entry point alone; this is taken into account when interpreting the operative-time and functional comparisons. In both groups the fixation construct consisted of two Kirschner wires (one lateral and one medial), and no additional wires were placed. Representative intraoperative fluoroscopy of the conventional crossed-pin construct is shown in Section 3.

### 2.5. Postoperative Management

Both groups followed an identical postoperative protocol. The elbow was immobilized in a well-padded above-elbow cast at approximately 90° of flexion. Patients attended routine outpatient follow-up with clinical and radiographic assessment. Kirschner wires were removed after radiographic evidence of union, typically between three and six weeks after surgery, although removal was delayed in selected patients until satisfactory union had been confirmed. Active elbow range-of-motion exercises were commenced after wire removal.

### 2.6. Outcome Measures

The primary endpoint was functional outcome at final follow-up assessed using the Flynn criteria [28]. The Flynn criteria assess the cosmetic (carrying-angle) and functional (elbow-motion) results by comparison with the contralateral uninjured elbow. Categorical Flynn grades were assigned according to the original criteria: each component was graded separately by the magnitude of loss (0–5°, excellent; 6–10°, good; 11–15°, fair; greater than 15°, poor), and the overall grade for each patient was determined by the poorer of the two component grades. All categorical outcomes reported in Section 2.7 were derived in this way. In addition, a composite Flynn score was calculated in degrees as the arithmetic sum of the loss of carrying angle and the loss of elbow motion relative to the uninjured side, with lower scores indicating better outcomes; this composite is expressed in degrees rather than in points and was used solely to permit analysis of a single continuous variable. The summed composite is an adaptation of the original criteria and has not been formally validated as an outcome instrument, and it was therefore reported alongside, and not in place of, the original Flynn grading. Functional assessment was performed by an orthopedic surgeon independent of the treating surgical team. Because the pin configuration is visible on postoperative radiographs, this assessor was independent but could not be blinded to the fixation technique; the potential for assessment bias in the subjective components of the Flynn criteria is acknowledged as a limitation. Secondary outcomes included operative time, postoperative Baumann angle, carrying angle, loss of carrying angle, elbow range of motion, loss of elbow motion, time to radiographic union, and postoperative complications. A preoperative neurological examination of the injured limb was documented in all patients, and iatrogenic ulnar nerve injury was defined as a new postoperative ulnar nerve deficit in a patient with normal preoperative ulnar nerve function. Time to union was defined as the number of days from surgery until bridging callus was present across at least three cortices on standard radiographs together with absence of fracture-site tenderness. Operative time was obtained from the institutional operating room information system and defined as the interval from induction of anesthesia until completion of cast application. Radiographic measurements were performed using PACS digital measurement tools (PACS sysyem by philips, Best, The Netherlands, version: Philips Image Management 15).

### 2.7. Statistical Analysis

Statistical analysis was performed using IBM SPSS Statistics version 30.0 (IBM Corp., Armonk, NY, USA). Continuous variables were assessed for normality using the Shapiro–Wilk test. Normally distributed variables are presented as mean ± standard deviation and were compared using the independent-samples *t*-test, with the corresponding 95% confidence intervals (CIs) for the mean difference obtained directly from the *t*-test output. Although the Flynn criteria are derived from an ordinal grading system, the composite Flynn score was treated as a continuous variable for the primary analysis in keeping with common practice in the supracondylar fracture literature; the categorical Flynn grade distribution is also reported (Section 2.7) to allow interpretation independent of this assumption. Categorical variables are presented as frequencies and percentages and were compared using the Chi-square test or Fisher exact test as appropriate. The Fisher exact test was used for sparse complication outcomes. Odds ratios (ORs) with 95% CIs are reported for complication outcomes where estimable; the Haldane–Anscombe correction was applied when a 2 × 2 table contained a zero cell, and OR confidence intervals were derived using the normal approximation on the log-odds scale. All tests were two-sided, and *p* < 0.05 was considered statistically significant. Functional (Flynn) outcome was the primary outcome; the secondary outcomes were analyzed without adjustment for multiple comparisons and are therefore regarded as exploratory. No a priori sample-size calculation was performed; in a post hoc consideration, 35 patients per group provided approximately 80% power at a two-sided alpha of 0.05 to detect a standardized between-group difference of about 0.67 standard deviations (Cohen d) for continuous outcomes, and the study was underpowered to detect differences in rare complications such as iatrogenic ulnar nerve injury.

## 3. Results

A total of 70 children met the study eligibility criteria and were included in the final analysis. Thirty-five patients underwent fixation using the modified medial para-olecranon technique, and 35 patients treated with conventional crossed-pin fixation were selected from 68 eligible patients treated with conventional crossed pinning during the same study period by computer-generated random sampling stratified by Gartland type (frequency matching).

Baseline demographic and fracture characteristics were comparable between groups (Table 1). Mean age was 6.7 ± 2.8 years in the modified medial para-olecranon group and 6.9 ± 2.6 years in the conventional crossed-pin group. There were no meaningful differences in sex distribution, injured side, or time from injury to surgery; the Gartland distribution was identical by design because the control group was frequency-matched to the modified group by Gartland type. Follow-up ranged from six to at least twelve months: 62 of the 70 patients (88.6%) were followed for at least twelve months and the remaining eight for six to nine months.

Mean operative time was significantly shorter in the modified medial para-olecranon group (47.49 ± 7.50 min) than in the conventional crossed-pin group (66.00 ± 8.19 min; mean difference, −18.51 min; 95% CI, −22.26 to −14.76; *p* < 0.001). Radiographic union was achieved in all patients. Mean time to union was comparable between groups (27.71 ± 3.94 vs. 28.34 ± 4.12 days; mean difference, −0.63 days; 95% CI, −2.55 to 1.29; *p* = 0.5023).

**Figure 3:**
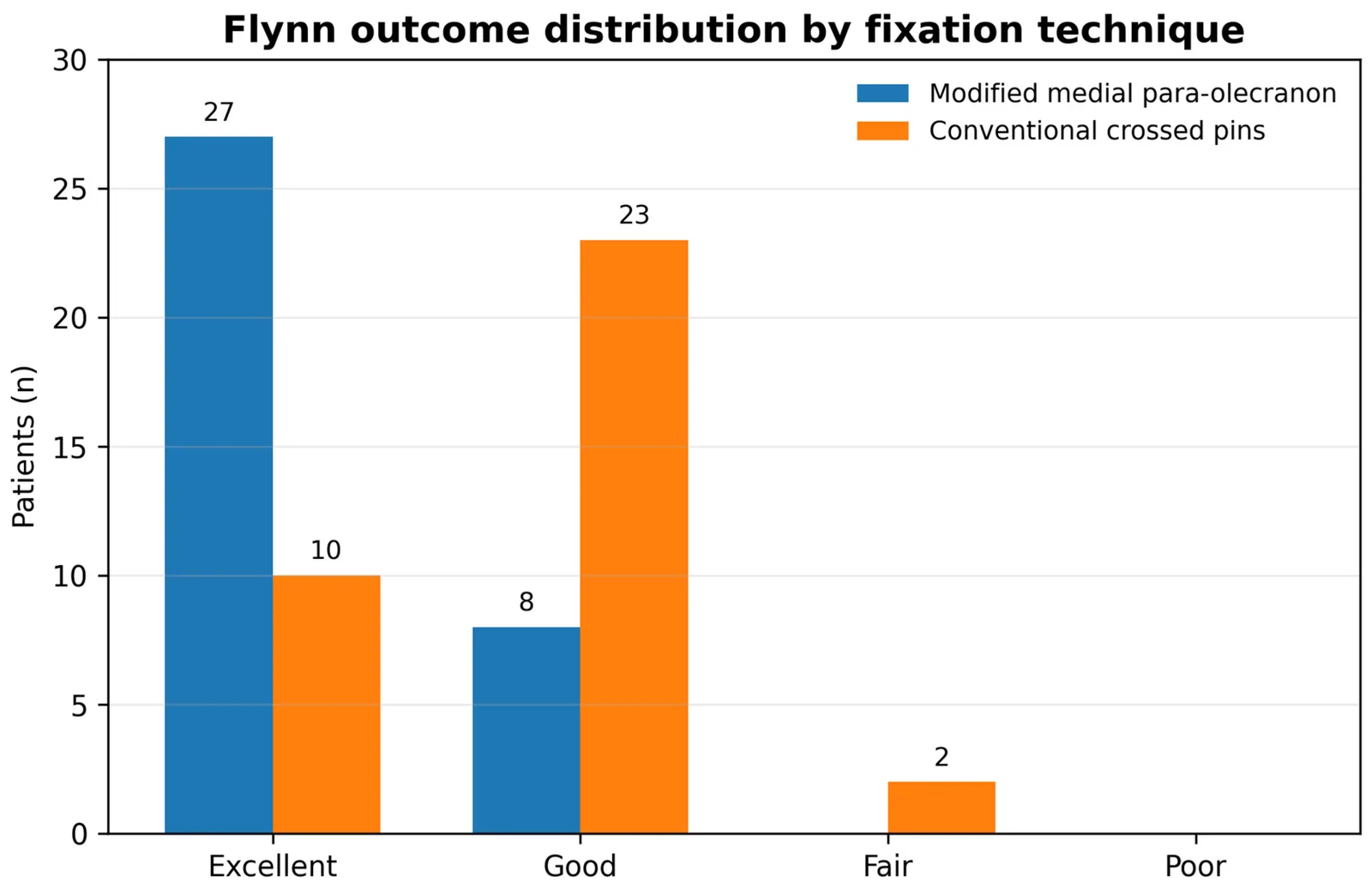
Distribution of Flynn outcome grades by fixation technique. The modified medial para-olecranon group had a greater number of excellent outcomes and fewer fair outcomes than the conventional crossed-pin group.

Functional outcomes are presented in Table 2 and Figure 3. Mean Flynn score was lower in the modified medial para-olecranon group (6.80 ± 3.31) compared with the conventional crossed-pin group (10.74 ± 3.70; mean difference, −3.94; 95% CI, −5.61 to −2.27; *p* < 0.001). Excellent Flynn outcomes were observed in 27 patients (77.1%) and 10 patients (28.6%), respectively.

Radiographic outcomes are summarized in Table 3. Postoperative Baumann angle was comparable between groups (mean difference, 1.32°; 95% CI, −0.46 to 3.10). Loss of carrying angle was lower in the modified medial para-olecranon group (2.17 ± 1.82°) than in the conventional crossed-pin group (3.09 ± 2.05°; mean difference, −0.92°; 95% CI, −1.82 to −0.02; *p* = 0.0453). Loss of elbow motion was also lower in the modified group (4.63 ± 3.12° vs. 7.66 ± 3.58°; mean difference, −3.03°; 95% CI, −4.63 to −1.43; *p* < 0.001).

**Figure 4:**
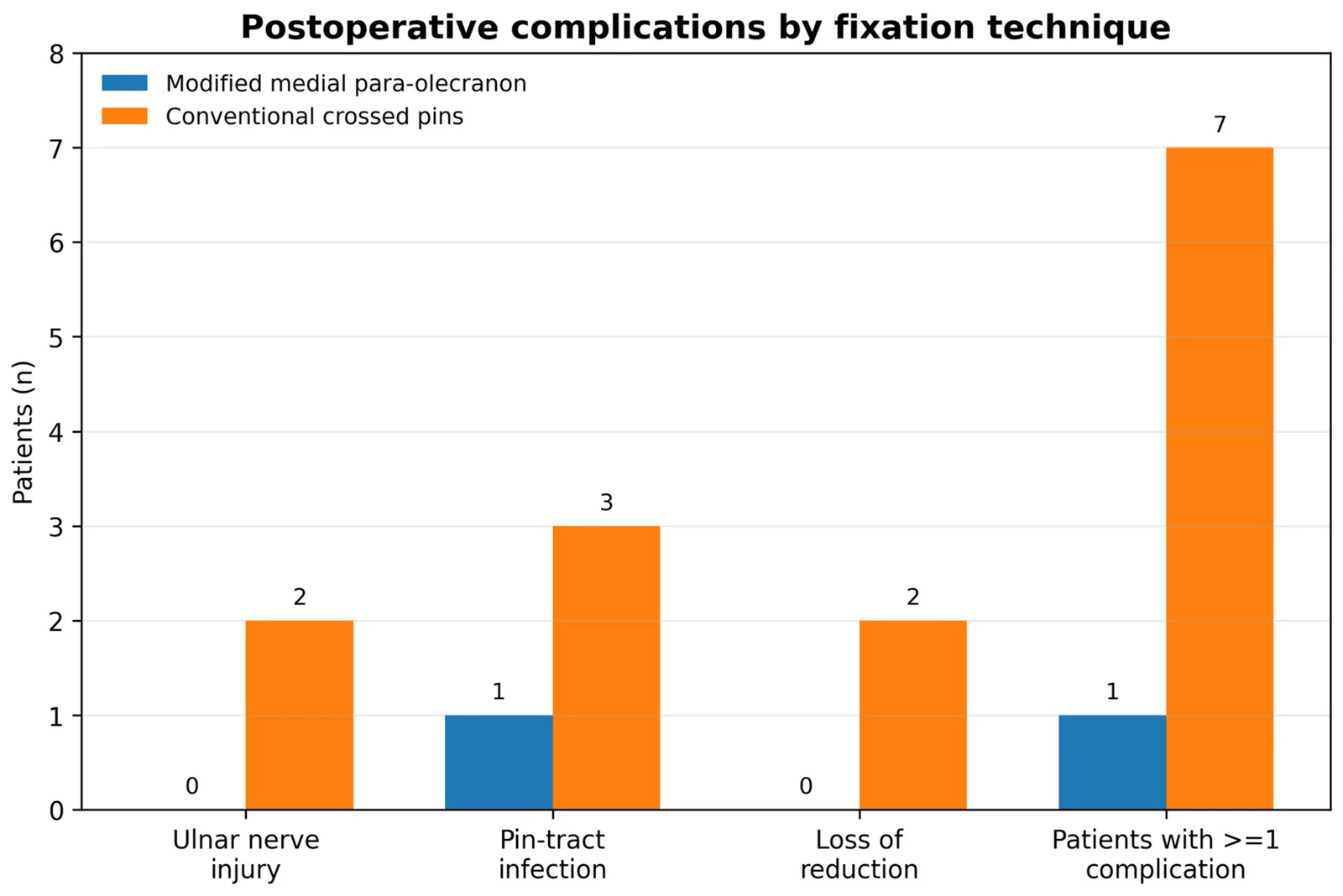
Postoperative complication summary by fixation technique.

**Figure 5:**
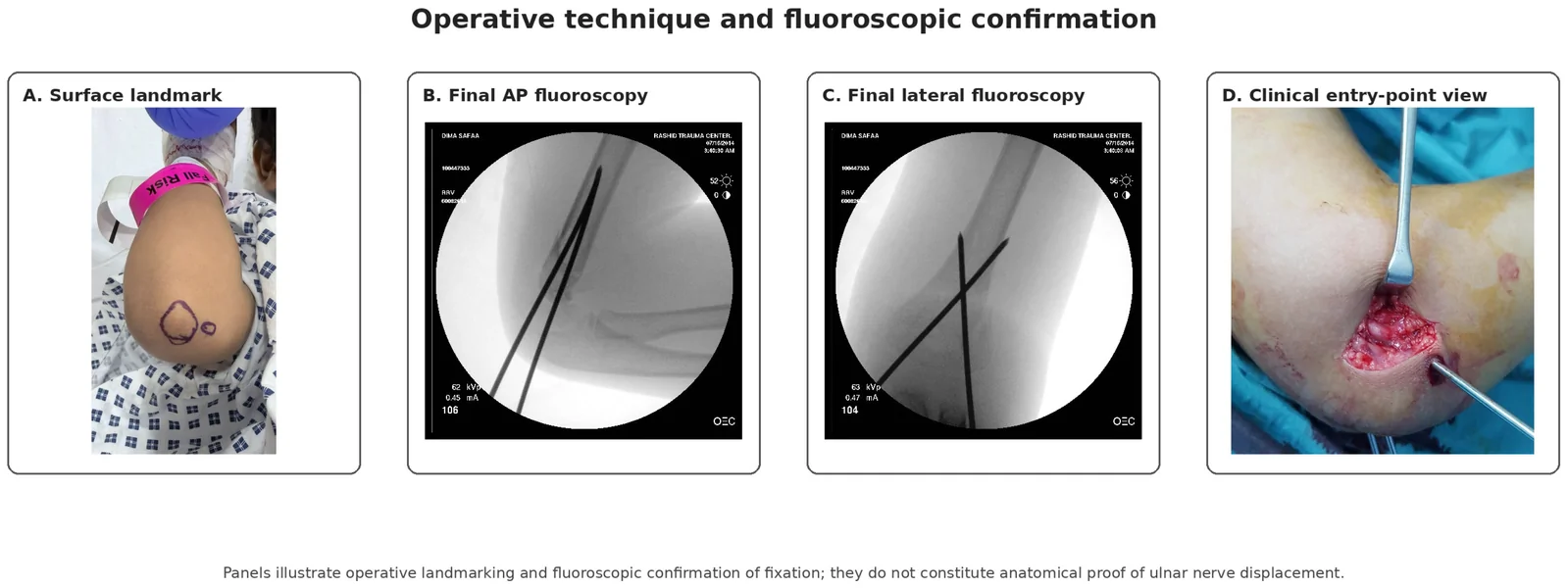
Operative technique and fluoroscopic confirmation. (**A**) Surface landmarking for the modified medial para-olecranon entry point; the patient’s face has been obscured for confidentiality. (**B**,**C**) Final fluoroscopic views confirming crossed-pin fixation and bicortical purchase. (**D**) Illustrative photograph demonstrating the position of the ulnar nerve relative to the para-olecranon entry point and wire trajectory; the modified technique was performed percutaneously in all study patients, and this exposure is shown for anatomical illustration only. The figure illustrates surgical landmarks and fixation confirmation only and does not depict routine use of a medial incision.

**Figure 6:**
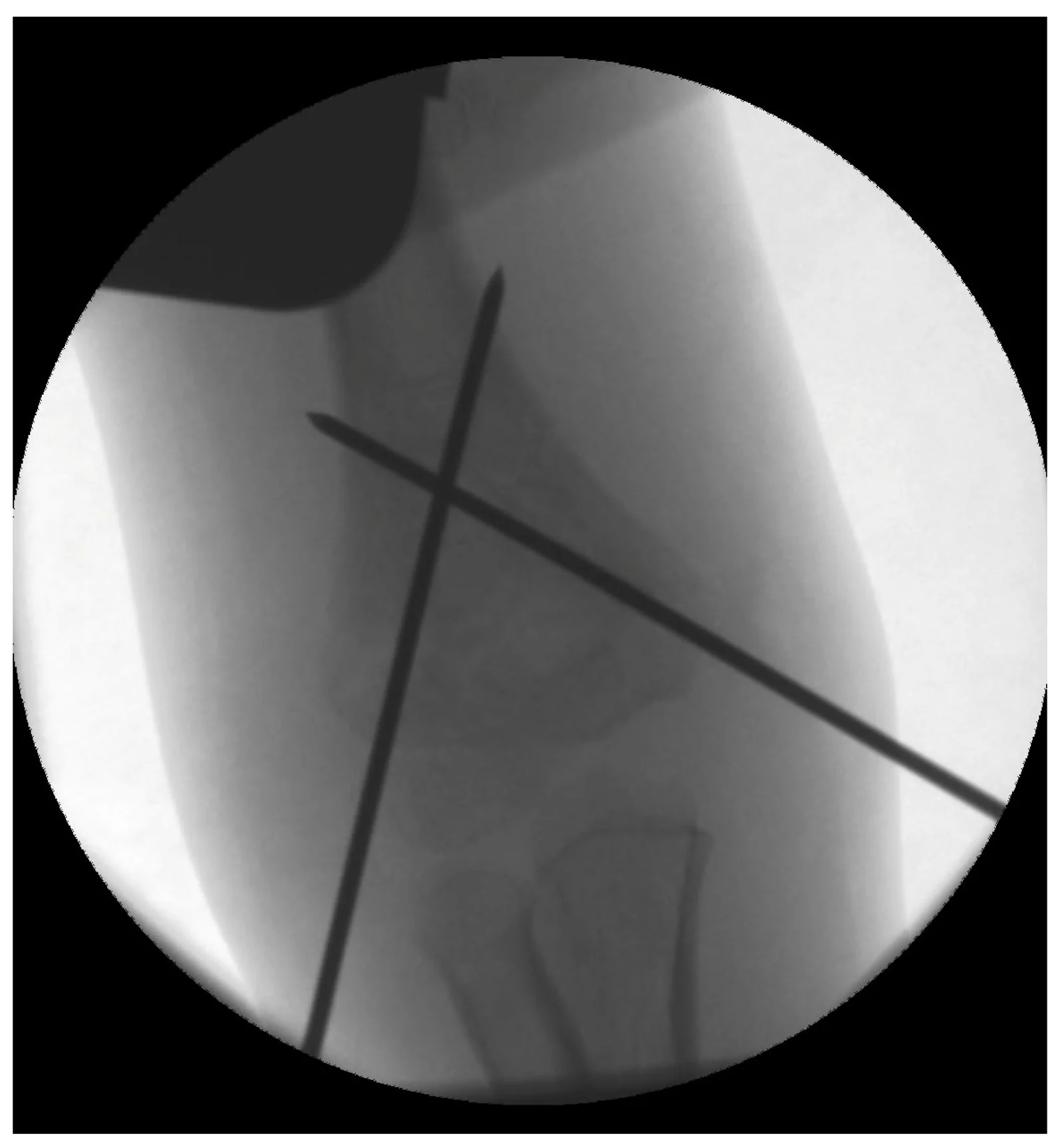
Representative intraoperative fluoroscopic image of the conventional crossed-pin technique, showing the crossed medial and lateral Kirschner wires spanning the supracondylar fracture with bicortical purchase. This complements the modified-technique fluoroscopy shown in Figure 5.

Postoperative complications are summarized in Table 4 and Figure 4. No postoperative iatrogenic ulnar nerve injuries were observed in the modified medial para-olecranon group. Two postoperative ulnar nerve injuries occurred in the conventional crossed-pin group (OR, 0.19; 95% CI, 0.01 to 4.08). Both occurred in conventional cases performed without a mini-open medial exposure; no ulnar nerve injury occurred among the 10 conventional cases in which a mini-open exposure was used, although these subgroups are too small to support any comparison. Both patients had documented normal preoperative ulnar nerve function; both deficits were transient sensory paraesthesia (neurapraxia) without motor involvement. Both were first documented on the first postoperative day. Neither patient underwent secondary surgical exploration of the nerve, the medial wire was not removed or repositioned for the deficit, and both resolved spontaneously with observation alone, with complete resolution documented at 112 and 144 days after surgery, respectively. Pin-tract infection occurred in one patient in the modified group and three patients in the crossed-pin group. Loss of reduction occurred in two patients in the crossed-pin group and was not observed in the modified group. Overall complications occurred in 1 patient (2.9%) in the modified medial para-olecranon group and 7 patients (20.0%) in the crossed-pin group (OR, 0.17; 95% CI, 0.03 to 1.02; Fisher exact *p* = 0.055). Operative landmarking and fluoroscopic confirmation are shown in Figure 5 and Figure 6.

## 4. Discussion

### 4.1. Principal Findings

This retrospective comparative cohort study evaluated the clinical performance of a modified medial para-olecranon technique for displaced pediatric supracondylar humerus fractures. The principal findings were that the modified medial para-olecranon technique was associated with better Flynn outcomes, shorter operative time, smaller losses of carrying angle and elbow motion, and fewer observed postoperative ulnar nerve injuries while maintaining comparable fracture union and postoperative Baumann angle. These findings suggest that changing the medial wire entry point may be a practical option for surgeons who prefer a crossed-pin construct in selected displaced supracondylar humerus fractures.

Importantly, this study evaluated clinical outcomes rather than the anatomical proximity of the wire to the ulnar nerve, which would require cadaveric or imaging demonstration: the absence of postoperative ulnar nerve injury in the modified medial para-olecranon group is clinically encouraging but does not prove a specific anatomical mechanism.

### 4.2. Context Within the Existing Literature

Closed reduction and percutaneous pinning is widely accepted for displaced Gartland type II, III, and IV supracondylar humerus fractures because it provides reliable stability with limited soft-tissue disruption [4,5,6,7,26,27,28]. The debate regarding optimal pin configuration has persisted for decades. Crossed medial-lateral pinning has repeatedly shown greater torsional and rotational stability in biomechanical studies [8,9,10]. In contrast, lateral-only fixation avoids medial pin insertion and therefore reduces the direct risk to the ulnar nerve, but concerns remain regarding rotational stability in selected unstable fractures [11,12,13,14,22].

Systematic reviews and meta-analyses have generally shown that both lateral-only and crossed-pin constructs can produce satisfactory clinical outcomes when applied correctly, but the trade-off between stability and iatrogenic ulnar nerve injury continues to influence surgeon decision-making [11,12,13,14,27,29]. The present study does not argue against lateral-only fixation. Instead, it addresses a narrower and clinically relevant question: whether a modification of the medial entry point can preserve the principles of crossed-pin fixation while maintaining favorable clinical performance.

### 4.3. Functional and Radiographic Outcomes

Functional recovery was better in the modified medial para-olecranon group, with a lower mean Flynn score and a higher proportion of excellent Flynn outcomes. These findings are relevant because Flynn criteria remain one of the most commonly used outcome measures after pediatric supracondylar humerus fracture fixation [28]. The differences in functional outcomes were accompanied by smaller losses of carrying angle and elbow motion, suggesting that the observed improvement was not limited to a single clinical parameter.

Radiographic outcomes were also reassuring. Postoperative Baumann angle and time to radiographic union were comparable between groups, indicating that modifying the medial entry point did not appear to compromise coronal alignment or fracture healing. This is important because the principal rationale for maintaining a crossed-pin construct is preservation of mechanical stability, especially in unstable fracture patterns [8,9,10,30,31]. The present findings therefore support the technical feasibility of the modified medial para-olecranon technique as a crossed-pin variant rather than a fundamentally different fixation construct.

The lower loss of carrying angle and elbow motion observed in the modified medial para-olecranon group should nevertheless be interpreted cautiously. Baseline characteristics were comparable and postoperative rehabilitation was standardized, but the retrospective design cannot exclude unmeasured confounding. Surgeon technique, fracture reducibility, intraoperative handling, swelling, and subtle differences in postoperative assessment may all influence final clinical outcome. Moreover, the magnitude of the differences in operative time and functional outcome is larger than a change in medial entry point alone would be expected to produce and probably reflects additional factors, including individual surgeon and possible era effects, that could not be fully controlled in this retrospective design; the modified technique should therefore be interpreted as a feasible and safe alternative rather than as proven to be superior. No multivariable adjustment for confounding was possible because individual allocation, operating surgeon, and year of surgery could not be reconstructed for each case; comparability rested only on the balanced baseline variables (age, sex, injured side, identical Gartland distribution, and time from injury to surgery). Residual confounding therefore cannot be excluded, and the functional and operative-time differences are best regarded as hypothesis-generating associations rather than demonstrated effects of the technique itself.

### 4.4. Operative Efficiency

Operative time was substantially shorter in the modified medial para-olecranon group. A shorter procedure may reflect easier identification of the medial entry point, less need for repeated wire repositioning, avoidance of additional soft-tissue dissection in many cases, or greater technical familiarity among surgeons using the modified approach. Similar concerns about operative workflow have been discussed in relation to safe medial pinning methods, including mini-open medial approaches and other protective strategies [18,19,32]. This distinction is directly relevant here: the conventional group required partial elbow extension in every case and a mini-open medial exposure in 10 of 35 cases, whereas the modified group was uniformly percutaneous and performed in hyperflexion. A substantial part of the operative-time difference, and potentially some of the functional difference, is therefore likely attributable to the surgical approach itself and to the different operating surgeons rather than to the medial entry point alone.

A strength of the operative time analysis is that time was extracted from the institutional operating room information system rather than estimated manually. However, the measured interval extended from anesthesia induction to cast completion, meaning it reflects total operating room procedural time rather than pure wire insertion time. Therefore, although the observed difference is clinically relevant, the study cannot determine which portion of the workflow accounted for the shorter duration.

### 4.5. Ulnar Nerve Injury and Medial Pinning

The neurological findings are clinically important but require careful interpretation. No postoperative iatrogenic ulnar nerve injuries were observed in the modified medial para-olecranon group. In the conventional crossed-pin group, two postoperative ulnar nerve injuries occurred, in conventional cases performed without a mini-open medial exposure. Both patients were neurologically intact before surgery, and both deficits were transient sensory paraesthesia (neurapraxia) that were first documented on the first postoperative day, required no secondary exploration, and recovered fully by 112 and 144 days, respectively. Published rates of iatrogenic ulnar nerve palsy after crossed pinning vary, and reported risk is influenced by elbow position, wire trajectory, soft-tissue swelling, surgeon experience, and use of a mini-open technique [11,15,16,17,18,19,22,29].

Because ulnar nerve injury is an uncommon complication, the current sample size is insufficient to establish definitive superiority for nerve safety. The corrected complication analysis showed fewer overall complications in the modified medial para-olecranon group, but this should be interpreted as a clinically relevant observation rather than a definitive statistical conclusion. The most defensible interpretation is that fewer postoperative ulnar nerve injuries were observed in this cohort, not that the technique eliminates or proves prevention of ulnar nerve injury.

### 4.6. Relationship to Existing Technical Modifications

Several techniques have been proposed to reduce ulnar nerve risk during medial pin insertion. These include placing the medial pin with the elbow in extension, using a mini-open approach, palpating or directly protecting the nerve, and modifying the medial wire trajectory [18,19,32,33]. Mini-open medial pinning has the advantage of direct visualization or protection of the ulnar nerve, but it may increase operative steps and soft-tissue dissection. Elbow extension during medial pin placement may reduce nerve tension or alter the nerve relationship to the medial epicondyle, but it can also make maintenance of reduction more difficult in some fractures.

The modified medial para-olecranon technique differs from these approaches because it changes the entry point while preserving the crossed-pin construct. It does not require special equipment and does not necessarily require a separate medial incision. This may explain its shorter operative time in the present cohort, although the study was not designed to isolate procedural factors. The technique should therefore be regarded as a potentially useful option within the broader group of safe medial pinning strategies rather than as a replacement for all established approaches.

### 4.7. Anatomical Considerations

The proposed anatomical explanation for the modified medial para-olecranon technique must be framed carefully. A posterior or para-olecranon entry point may theoretically alter the spatial relationship between the advancing Kirschner wire and the ulnar nerve, and the schematic in Figure 2 should be read as an illustration of osseous landmarks and intended wire trajectory rather than as evidence of ulnar nerve displacement. However, this study did not evaluate ulnar nerve position using ultrasonography, magnetic resonance imaging, cadaveric dissection, or intraoperative nerve mapping, and therefore cannot confirm that the observed clinical findings were caused by a change in nerve position. This caution is reinforced by ultrasound studies showing that pediatric ulnar nerve anatomy varies during elbow motion and that anterior translation or subluxation during flexion does not occur uniformly in all children [20,21]; computational work on Kirschner-wire trajectory likewise indicates that pin position matters but cannot establish the patient-specific anatomical safety of any single technique [34]. The present study should therefore be cited as clinical outcome evidence, not as anatomical validation of the proposed mechanism.

### 4.8. Strengths

This study has several strengths. It included all consecutive patients treated with the modified medial para-olecranon technique during the study period, reducing selective inclusion within that cohort. The comparison group was randomly selected from a larger eligible cohort using the same inclusion and exclusion criteria. Baseline demographic and fracture characteristics were comparable, and both techniques were used during the same overall study period. The study also assessed multiple clinically relevant outcomes, including functional recovery, radiographic alignment, fracture union, operative time, and postoperative complications.

A further strength is the deliberate separation of clinical findings from mechanistic explanation: clinical outcomes are reported while the proposed anatomical mechanism is explicitly acknowledged as unproven, which is important in surgical technique studies where the technical rationale is often plausible but not directly measured.

### 4.9. Limitations

Several limitations should be considered. First, the retrospective single-center design introduces potential selection bias and limits generalizability. Second, treatment allocation was determined by routine clinical practice rather than randomization, so unmeasured confounding cannot be excluded. Third, because allocation was based on surgeon preference and expertise rather than randomization, confounding by indication cannot be excluded; although both techniques were used across the same overall study period by experienced consultants, the temporal distribution of cases within that period was not analyzed, so era-related changes in perioperative care and surgeon-level differences in technique or case selection may also have contributed to the observed differences in operative time and outcome. Fourth, the study compared the modified medial para-olecranon technique with conventional crossed pinning and did not include a lateral-only fixation cohort; therefore, the findings should not be interpreted as evidence against lateral-only pinning. Fifth, the sample size was modest, particularly for rare complications such as ulnar nerve injury, and no prospective sample-size calculation was performed. Because allocation, operating surgeon, and year of surgery could not be retrieved per case, no adjustment for these potential confounders was possible; the comparative functional, motion, and operative-time findings should therefore be regarded as hypothesis-generating associations rather than confirmed differences attributable to the technique.

Sixth, the anatomical mechanism proposed for the modified medial para-olecranon technique was not directly evaluated. No dynamic ultrasound, MRI, cadaveric analysis, or intraoperative nerve mapping was performed. Seventh, the outcome assessor, although independent of the treating surgical team, could not be blinded to the fixation technique because the pin configuration is apparent on postoperative radiographs, which may introduce assessment bias in the subjective components of the Flynn criteria. In addition, the continuous composite Flynn score used in this study is a summed adaptation expressed in degrees and is not a formally validated outcome instrument; the categorical Flynn grades reported in Table 2 were, however, assigned using the original Flynn criteria. Eighth, neurological outcomes were based on clinical documentation rather than routine nerve conduction studies, and postoperative Baumann and carrying angles were reported as absolute values without a contralateral uninjured-side comparison. Finally, although most patients were followed for at least one year, assessment focused on fracture healing and early functional recovery; longer-term remodeling, late deformity, and patient-reported outcomes were not systematically assessed.

### 4.10. Clinical Implications and Future Research

For surgeons who prefer crossed-pin fixation because of its mechanical stability, the modified medial para-olecranon technique may represent a feasible alternative medial entry strategy. The technique preserves the basic crossed-pin construct while modifying the site of medial wire insertion. In the present cohort, it was associated with favorable functional outcomes, shorter operative time, and fewer observed ulnar nerve injuries while maintaining radiographic alignment and fracture healing.

Future studies should proceed in two directions. First, prospective multicenter comparative studies with larger cohorts are needed to confirm clinical outcomes and complication rates. Second, dedicated anatomical studies using dynamic ultrasonography, MRI, cadaveric methods, or intraoperative imaging are required to determine whether the modified trajectory changes the relationship between the medial wire and the ulnar nerve. Only after both clinical and anatomical validation can broader recommendations be made.

## 5. Conclusions

In this retrospective comparative cohort, the modified medial para-olecranon technique was associated with favorable functional outcomes, shorter operative time, and fewer observed postoperative ulnar nerve injuries while maintaining comparable fracture union and radiographic alignment compared with conventional crossed pinning. The technique appears to be a feasible alternative medial pin insertion strategy for surgeons who prefer crossed-pin fixation. Because treatment was not randomized and potential confounders could not be adjusted for, the observed functional and operative-time differences should be considered hypothesis-generating associations rather than evidence that the technique is causally superior. Because the anatomical mechanism was not directly investigated and the study design was retrospective, prospective multicenter clinical studies and dedicated anatomical validation are required before definitive recommendations can be made.

## Figures and Tables

**Table 1:** Baseline demographic and fracture characteristics.

Characteristic	Modified Medial Para-Olecranon (*n* = 35)	Conventional Crossed Pins (*n* = 35)	*p*-Value
Age (years), mean ± SD	6.7 ± 2.8	6.9 ± 2.6	0.758
Male sex, *n* (%)	22 (62.9%)	20 (57.1%)	0.808
Female sex, *n* (%)	13 (37.1%)	15 (42.9%)	—
**Injured side**			**0.810**
Right, *n* (%)	16 (45.7%)	18 (51.4%)	—
Left, *n* (%)	19 (54.3%)	17 (48.6%)	—
**Gartland classification**			**1.000**
Type II, *n* (%)	14 (40.0%)	14 (40.0%)	—
Type III, *n* (%)	19 (54.3%)	19 (54.3%)	—
Type IV, *n* (%)	2 (5.7%)	2 (5.7%)	—
Time from injury to surgery (hours), mean ± SD	18.3 ± 9.7	19.1 ± 10.2	0.738

Note. Values are presented as mean ± SD or number (%). SD, standard deviation. *p*-values are shown for between-group comparisons where applicable.

**Table 2:** Operative and functional outcomes.

Outcome	Modified Medial Para-Olecranon (*n* = 35)	Conventional Crossed Pins (*n* = 35)	Mean Difference *	95% CI	*p*-Value
Operative time (min), mean ± SD	47.49 ± 7.50	66.00 ± 8.19	−18.51	−22.26 to −14.76	<0.001
Flynn score (°), mean ± SD	6.80 ± 3.31	10.74 ± 3.70	−3.94	−5.61 to −2.27	<0.001
Flynn grade distribution	—	—	—	—	<0.001
Excellent, *n* (%)	27 (77.1%)	10 (28.6%)	—	—	—
Good, *n* (%)	8 (22.9%)	23 (65.7%)	—	—	—
Fair, *n* (%)	0 (0%)	2 (5.7%)	—	—	—
Poor, *n* (%)	0 (0%)	0 (0%)	—	—	—

Note. Values are presented as mean ± SD or number (%). * Mean difference is modified medial para-olecranon minus conventional crossed pins. CI, confidence interval; SD, standard deviation. Flynn grade distribution *p*-value refers to the overall categorical distribution.

**Table 3:** Radiographic outcomes and fracture union.

Outcome	Modified Medial Para-Olecranon (*n* = 35)	Conventional Crossed Pins (*n* = 35)	Mean Difference *	95% CI	*p*-Value
**Alignment**					
Baumann angle (°), mean ± SD	71.63 ± 3.50	70.31 ± 3.93	1.32	−0.46 to 3.10	0.1146
Carrying angle (°), mean ± SD	11.23 ± 2.14	10.86 ± 2.31	0.37	−0.69 to 1.43	0.4872
**Motion**					
Loss of carrying angle (°), mean ± SD	2.17 ± 1.82	3.09 ± 2.05	−0.92	−1.82 to −0.02	0.0453
Loss of elbow motion (°), mean ± SD	4.63 ± 3.12	7.66 ± 3.58	−3.03	−4.63 to −1.43	<0.001
**Fracture union**					
Time to radiographic union (days), mean ± SD	27.71 ± 3.94	28.34 ± 4.12	−0.63	−2.55 to 1.29	0.5023

Note. Values are presented as mean ± SD. * Mean difference is modified medial para-olecranon minus conventional crossed pins. CI, confidence interval; SD, standard deviation.

**Table 4:** Postoperative complications.

Complication	Modified Medial Para-Olecranon (*n* = 35)	Conventional Crossed Pins (*n* = 35)	OR (95% CI) †	*p*-Value *
**Neurological**				
Iatrogenic ulnar nerve injury, *n* (%)	0 (0%)	2 (5.7%)	0.19 (0.01 to 4.08)	0.493
**Infectious**				
Pin-tract infection, *n* (%)	1 (2.9%)	3 (8.6%)	0.40 (0.06 to 2.90)	0.614
**Mechanical**				
Loss of reduction, *n* (%)	0 (0%)	2 (5.7%)	0.19 (0.01 to 4.08)	0.493
**Other**				
Compartment syndrome, *n* (%)	0 (0%)	0 (0%)	Not estimable	—
Vascular injury, *n* (%)	0 (0%)	0 (0%)	Not estimable	—
**Overall**				
Patients with ≥1 complication, *n* (%)	1 (2.9%)	7 (20.0%)	0.17 (0.03 to 1.02)	0.055

Note. * Fisher exact test was used for sparse categorical comparisons. † Odds ratios compare the modified medial para-olecranon group with the conventional crossed-pin group. ORs and 95% CIs were calculated using the Haldane–Anscombe correction when a 2 × 2 table contained a zero cell. CI, confidence interval; OR, odds ratio.

## Data Availability

The data presented in this study are available on request from the corresponding author because they were obtained from institutional electronic medical records and PACS and are subject to institutional governance, privacy, and ethical restrictions; access requires institutional and IRB approval.
